# Editorial: Biomechanics, technology, and athletic performance: pathways to sustainable health

**DOI:** 10.3389/fspor.2026.1933614

**Published:** 2026-08-05

**Authors:** Jian-Zhi Lin, Akihiro Tamura, Hsien-Te Peng, Wei-Hsun Tai

**Affiliations:** 1 Department of Physical Education, National Taiwan University of Sport, Taichung, Taiwan; 2 Research Institute for Sport Science, Nippon Sport Science University, Tokyo, Japan; 3 Department of Physical Education, Chinese Culture University, Taipei, Taiwan; 4 College of Forestry, Fujian Agriculture and Forestry University, Fuzhou, China

**Keywords:** biomechanics, health promotion, injury, movement analysis 3, wearable - user interaction

## 1 Introduction

This Research Topic, hosted under the “*Biomechanics and Control of Human Movement*” section of Frontiers in Sports and Active Living, establishes a critical intersection where movement science, emerging digital technologies, and long-term athletic longevity converge. Biomechanics has officially spilled over the laboratory walls; over the past two decades, the discipline has aggressively integrated engineering, muscle physiology, and machine learning to push assessment out of tightly controlled facilities and directly into active training fields, clinical hubs, and adaptive sports settings. Compiling 15 multidisciplinary articles, this Research Topic spans elite sports monitoring, wireless sensing, neuromuscular control, geriatric rehabilitation, and customized assistive design to demonstrate how modern human movement science supports “sustainable health” a paradigm shifting the focus from short-term podium performance to maintaining functional capacity across the full lifespan (Al Attar, Mahdi et al., Chen et al., Duan et al., Legnani et al., Zheng et al.). Ultimately, technological precision is no longer treated as an isolated end goal; instead, these contributions operationalize raw biomechanical data into scalable, equitable, and prospective tools designed for real-world injury reduction and intelligent clinical decision support.

## 2 Digital translation: scaling beyond laboratory boundaries

The transition from marker-based optical capture to accessible field technology is the most pressing technical shift in modern motion analysis. Rather than replacing the laboratory gold standard, these emerging tools democratize it.

- Markerless and monocular innovations

Three-dimensional joint tracking no longer requires multi-camera laboratory arrays or extensive calibration:

Iizuka et al. solved a major barrier in remote assessment by applying geometry-informed correction algorithms to browser-based monocular capture, successfully driving projection errors down to clinically acceptable thresholds during squats.

Pan et al. tackled a historically difficult cohort by deploying the markerless OpenCap platform to track lower-limb loading in preschool children on a balance beam. Their data revealed that early asymmetrical joint loading reflects developing neuromuscular control rather than underlying pathology, proving that complex pediatric screening can now bypass laboratory resistance.

- Continuous workload and on-field tracking

Wearable inertial sensors (IMUs) and surface electromyography (sEMG) have shifted assessment from isolated snapshots to continuous seasonal surveillance:

Havanecz et al. tracked elite youth soccer players across an entire competitive season using IMUs. They discovered that movement asymmetries are not static unilateral traits but are strictly dependent on the interplay between leg dominance and tactical direction insights blind to single-day lab tests.

Chen et al. translated this approach into Ultimate Frisbee, coupling wearable monitoring with neuromuscular interventions to build individualized mechanical workload thresholds that directly suppress injury spikes.

Mahdi et al. took IMUs and sEMG into para-fencing, demonstrating that elite offensive strikes depend heavily on trunk-stabilization timing and muscle mechanical properties rather than raw upper-limb velocity alone.

- Digital design and kinetic mapping

Computational modeling is reshaping orthotics and multi-dimensional analysis:

Chhikara et al. challenged digital manufacturing workflows by evaluating whether traditional anatomical palpation remains mandatory in fully digital foot orthotic design, reinforcing that software must embed, rather than replace, clinical intuition.

Shi et al. moved beyond traditional discrete variables by applying Statistical Parametric Mapping (SPM) to study arch-support insoles during countermovement jumps. Continuous analysis mapped temporal kinetic shifts across propulsion and landing, isolating precisely when the insole enhances joint stability.

## 3 Clinical translation, rehabilitation, and lifelong performance

Athletic performance and musculoskeletal longevity are interconnected facets of optimized motor control. The articles here treat human movement as an adaptable biological system.

- Technical adaptability and assistive design

Elite performance requires the dynamic reorganization of mechanics under changing environmental constraints:

Chida et al. quantified how target distance changes lower-limb kinematics in elite fencing lunges, proving that long-distance accuracy relies on magnified rear-leg joint excursions to generate center-of-mass velocity.

Ishikawa et al. benchmarked AI-assisted judging against human expert evaluation during the complex gymnastics Switch Leap to Ring Position, isolating the definitive kinematic markers that human judges use to recognize technical mastery.

Maikos et al. advanced adaptive engineering by designing and validating a customized golf prosthesis. The mechanical linkage enabled a veteran with bilateral upper-limb loss to execute a fluid, independent golf swing, linking biomechanical restoration directly to quality of life.

- Standardized loading and neuromuscular medicine

Refining clinical protocols and understanding holistic movement patterns is essential for active aging and pathology management:

McGinley et al. exposed how fragile clinical testing can be: subtle variations in squatting verbal cues significantly altered lower-extremity kinematics and joint loading in adolescents with femoroacetabular impingement syndrome. This emphasizes the urgent need for rigid, standardized clinical assessment definitions.

Duan et al. dissected the mechanics of Tai Chi (Xuling Dingjin posture), proving it triggers earlier deep lumbar stabilizer activation, dampens spinal rotation, and homogenizes disc loading during stair descent—offering a clear mechanical explanation for fall risk reduction in older adults.

Zheng et al. paired postural tracking with respiratory analysis in experienced Tai Chi practitioners, identifying an adaptive coupling between breathing rhythms and lower-limb mobility that optimizes sensorimotor control.

Legnani et al. tracked real-world outcomes after unicompartmental knee arthroplasty, showing that successful joint restoration allows the majority of active individuals to safely return to recreational sports, validating surgery through sustained lifelong physical activity.

## 4 Future directions: toward predictive and proactive frameworks

The current state of the art excels at description and simulation; the next stage requires a shift toward prospective, intelligent, and multimodal integration.

Multimodal Data Fusion: Future research must bridge separate tracking systems. Kinematics, kinetics, and neuromuscular activation should be compiled into single analytical engines to assess how fatigue and skill acquisition interact in real time.

True Longitudinal Surveillance: Cross-sectional designs only capture a single moment. Biomechanics must prioritize multi-month wearable surveillance to capture subtle mechanical adaptations before micro-trauma converts into structural injury.

- AI as an active decision companion:

Al Attar laid the groundwork with the WASe-∞ framework, a computational model integrating physiological, neuromuscular, and cognitive strain to predict motor intent collapse under stress.

Future AI must transition from these descriptive simulations into live, prospective decision-support tools. These tools should provide coaches and physical therapists with real-time risk mitigation and automated adjustments for training volume.

Inclusive Customization: Digital manufacturing and markerless screening must target clinical equity, ensuring custom prosthetics, orthotics, and pediatric motor screening are accessible outside elite facilities.

## 5 Conclusions

Biomechanics is no longer an observational science confined to laboratory force plates and marker setups; it has matured into a translational platform linking technological innovation directly to clinical application, personalized intervention, and public health. By embedding markerless tracking, wearable analytics, and machine learning into fields and clinics, this Research Topic offers critical new perspectives on optimizing performance, mitigating injury risks, and safeguarding human movement competence across the lifespan—from pediatric development to geriatric functional recovery. We extend our sincere gratitude to the authors, reviewers, and editorial team whose rigorous contributions made this Research Topic possible. Ultimately, these 15 articles break down traditional disciplinary silos, and we expect these insights to catalyze future cross-disciplinary collaborations that will drive the continuous evolution of biomechanics and human movement control toward a more active, sustainable society.

